# Between stability and change: Tensions in the Norwegian electric mobility transition

**DOI:** 10.1177/03063127211022842

**Published:** 2021-07-10

**Authors:** Martin Anfinsen

**Affiliations:** The Norwegian University of Science and Technology (NTNU), Trondheim, Norway

**Keywords:** electric vehicles, sustainable transitions, multi-level perspective, actor-network theory, electric mobility

## Abstract

Norway, where a majority of new cars sold are currently electric, has emerged as a rich location for studying transitions towards electric mobility. Such transitions have often been conceptualized through a Multi-Level Perspective (MLP), which generally designates the technology as a disruptive niche with potential to upend the obdurate and problematic automobilty regime. Drawing upon Actor-Network Theory (ANT), this article re-examines this designation and provides nuance to theories of sustainable transition. This change in perspective enable us to re-centre user practices and investigate how electric vehicle drivers operate within complex human/non-human networks. Rather than viewing stability and change as the result of interactions between pre-determined levels, ANT allows us to explore how stability and change is co-produced in a multitude of locations. Drawing upon qualitative interview data, the article finds that new configurations of users and technology are currently emerging, elucidating dynamics of sociotechnical change, while cultural and geographical barriers to more radical mobility shifts are equally pronounced. As such, electric mobility currently finds itself between reinforcing the automobility system, while also engendering exciting new associations between drivers, cars and the world outside the windshield.

The automobile introduced unseen levels of mobility, but is associated with environmental harm, urban sprawl, stifling congestion and the displacement of pedestrians and cyclists. Industrial, cultural and economic dependencies have made automobile-based transportation systems exceedingly durable, while influencing how our societies are structured and how we structure our individual lives (Sheller and Urry, 2000; Urry, 2004). Thus, the automobile stands as a ‘literal iron cage of modernity’ (Urry, 2004: 28).

In the face of a problematic, yet stable, system of automobility, the environmental benefits and growth potential of electric vehicles (EVs) has attracted enthusiasm. With few local emissions, and significantly reduced overall emissions, electric vehicles are emerging as a potential solution to environmental issues stemming from the fossil-fuelled automobility system (Ingeborgrud and Ryghaug, 2017; Mitchell et al., 2010; Sovacool, 2017). The disruptive qualities of electric vehicles, combined with steadily rising global EV sales, has led several scholars to categorize the EV as an emerging niche with the potential to challenge the established fossil fuel-based automobility regime (Dijk et al., 2013, 2016; Figenbaum, 2017; Ryghaug and Skjølsvold, 2019; Whittle et al., 2019). At the same time, electric vehicles are bound to resource-intensive methods of production, and the technology do not appear to solve the *other* challenges associated with automobility, as formulated by Urry (2004).

In what ways, then, does the EV represent an alternative to the established system of automobility? Alternatively, does it rather serve as an extension of the system, while solving specific issues pertaining to local emissions?

**Figure 1.**
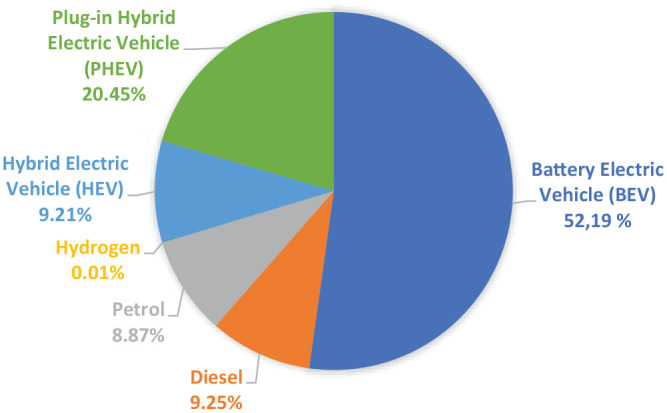
Registered passenger cars in Norway 2020, excluding used imports.^a^ ^a^Adapted and translated from The Norwegian Electric Vehicle Association (2020), and ‘Opplysningsrådet for Veitrafikken’ (Information Council for the Road Traffic). Last updated 30 November 2020.

In this article I investigate these questions empirically, looking at users’ representations of EV mobility in the Norwegian context. In Norway, where 98% of the energy consumed stems from renewable sources (Norwegian Energy Regulatory Authority, 2019), the environmental benefits of EVs have been widely lauded. Sales of EVs in Norway have grown through the 2010s, thanks to substantial government incentives, rising environmental concern and technological developments (Ryghaug and Skjølsvold, 2019). In 2020, more than two-thirds of new cars sold in Norway were either Battery Electric Vehicles (BEVs) or Plug-in Hybrid Vehicles (PHEVs) (Norwegian Electric Vehicle Association, 2020); in this article I treat both BEVs and PHEVs as EVs, but I specify when PHEVs are at issue. The prevalence of EVs makes Norway a compelling site for studying the transition towards electromobility, while simultaneously raising the question of what may be learned from this relatively unique geographical, economic, cultural and political setting. Though I focus on the specificities of the Norwegian transportation transition, implications can be drawn for other contexts, as I discuss in my conclusion.

The article builds on qualitative interviews with EV-owning households, analysed with theories developed to study sociotechnical sites and the production of stability and change (Latour, 1987; Pinch and Bijker, 1984). Studies of sustainable transformation in the transportation system often use a Multi-Level Perspective (MLP) to theorize these dynamics. Existing regimes, such as automobility, have stability in these models, while sources of substantive change exist in niches outcompeting the regime via openings enabled by pressures from the overarching landscape level (Geels, 2002; Rip and Kemp, 1998). In contrast, I investigate dynamics of change and stability with an Actor-Network Theory (ANT) approach, eschewing hierarchically oriented levels by adopting a flat ontology (Latour, 2005) to focus on practices, technologies and users. This article’s aim is twofold: (1) to investigate the sociotechnical systems and actor-networks in which EVs are embedded, with a specific focus on relations between actors, technologies and practices, and (2) to enrich the understanding of heterogenous actor constellations and assemblages crucial to sustainable transitions, while illustrating how stability and change is co-produced in a multitude of locations.

## Levelling the levels: From MLP to ANT

Sheller and Urry (2000) and Urry (2004) propose a systemic perspective on automobility, emphasizing the interplay between technical, social and cultural dimensions of automobility, rather than insular technology (the car) and users (drivers). This system is described as highly resistant to change, given the breadth and variance of users and stakeholders constituting the system, myriad path dependences or lock-ins, and how our societies have grown around and adapted to car technology (Sheller and Urry, 2000; Urry, 2004). Through highlighting the *system* of automobility, they argue that the dominance of automobility in the 20th century and beyond is ‘the single most important cause of environmental resource-use’ (Urry, 2004: 26).

Given the variance in the complex sociotechnical elements of this highly stable system, the societal challenges posed by automobility have generated several studies employing MLP (Geels, 2002; Geels and Schot, 2007; Rip and Kemp, 1998), which has advanced sustainability transition research (Köhler et al., 2019). MLP takes a systemic and socio-technical perspective on mobility and organizes the complex systems into three levels; the regime, landscape and niche (Geels, 2002), where a transition denotes the movement from one sociotechnical regime to another (Geels and Schot, 2007). While MLPs enable analyses of how transitions occur in the interplay between these levels, the general dynamic is that:

(a) niche-innovations gradually build up internal momentum, (b) niche-innovations and landscape changes create pressure on the system and regime, and (c) destabilization of the regime creates windows of opportunity for niche-innovations, which then diffuse and disrupt the existing system. (Geels, 2019: 190)

When analysing mobility transitions, the automobility system, as described by Urry (2004), is conventionally characterized as the dominant regime in need of change (Dijk et al., 2013; Geels, 2012). Here, EVs represent an emerging niche against the magnitude and historical obduracy of the regime.

MLP has been influential as a result of the combination of clarity, sociotechnical depth and ease of use, providing a ready-made template to frame complex empirical findings on sustainable transitions. Sovacool and Hess (2017) highlight MLP as the most popular model in a substantial cross-cutting and cross-disciplinary ordering of theories on sociotechnical change (Sovacool and Hess, 2017), and the diffusion of electric vehicles around the turn of the century constituted an early test case for the emerging theory (Schwanen, 2016). Consequently, MLP has been central in analysing transitions towards electromobility.

Several scholars have, however, critically interrogated the perceived lack of actors and agency in MLP-oriented studies (Genus and Coles, 2008; Hommels et al., 2007; Shove and Walker, 2007; Skjølsvold et al., 2018; Smith et al., 2005). Additionally, the theory has been criticized for missing the politics of everyday life (Shove and Walker, 2007), and drawing vague demarcations of niches, regimes and landscapes (Berkhout et al., 2004; Wells and Lin, 2015). Lastly, it is argued that while MLP is a powerful tool for understanding change, there has been ‘a relative neglect of how embedded regimes may have diverse evolutionary pathways including non-change’ (Steinhilber et al., 2013: 532). These criticisms have led to more comprehensive models, and more nuanced perspectives on transitions (Geels, 2011, 2019; Papachristos, 2018). When it comes to the Norwegian electro-mobile transition in particular, recent promise has been found in the geographic, temporal and relational complexities of what has been characterized as regime-led transition (Skjølsvold and Ryghaug, 2020).

Other sociotechnical theories have, however, been employed to supplement MLP. Sheller (2012), for instance, adds a cultural inflection to the approach, demonstrating how automobility is shaped by cultural factors, but also how culture is shaped by technology. Such mobility cultures are deemed as significant as regimes and landscapes in reinforcing the stability of automobility systems, while Sheller adds that the ‘phenomenology of car use highlights the driving body and the passenger as assemblages of social practices, embodied dispositions, emotional orientations and physical affordances which produce a “tacit automobilized embodiment”’ (Sheller, 2012: 197). Here, Sheller explicitly draws from Actor-Network Theory (ANT), emphasizing the interconnectedness of driver, car and the physical environment that is being traversed. In contrast to the nested and hierarchical ordered levels of MLP, ANT offers a flat ontology (Latour, 2005). Rather than viewing stability and change as an outcome of the interplay between obdurate regimes and emerging niches, ANT allows us to explore how stability and change is rather co-produced in a multitude of locations.

The concept of ‘assemblage’ (Latour, 2005) is central to ANT-oriented mobility research, referring to ‘the ordering of dissimilar entities so that they work together towards a common goal for a particular amount of time’ (Sovacool, 2017: 82). Here, ‘assemblage’ denotes the heterogeneous socio-material elements of the network, and how they coalesce into specific objects or phenomena. From this perspective, a driver and her car can, for instance, be understood as a driver-car: ‘a form of social being that produces a range of social actions that are associated with the car; driving, transporting, parking, consuming, polluting, killing, communicating and so on’ (Dant, 2004: 61f). To understand automobility from this perspective, we need to map the socio-material entanglements between driver and technology and how this assemblage operates and relates to the natural and man-made landscape. More generally, ANT has been highlighted by Sovacool (2017) as a useful approach for modelling mobility transitions, and by Genus and Coles (2008: 1443) as way of supplementing MLP by ‘exposing the processes of negotiation and enrolment that actors engage in to join a network facilitative of technology–society development’. This is particularly pertinent as the issue of agency within the frames of MLP are yet to be ‘solved’ (Geels, 2011; Papachristos, 2018). Taken together, the ubiquity of MLP in mobility transition research, its blind spots, and the potential for ANT to supplement these accounts, makes a compelling argument for a constructive juxtaposition of these perspectives.

Previous studies on EVs from a sociotechnical perspective have employed different foci and a variety of methods. These have tended to focus on change over stability, revealing the transformative potential of electric mobility. Studies include changes in specific user scripts (Gjøen and Hård, 2002), user perceptions (Ingeborgrud and Ryghaug, 2017), gender and identity (Anfinsen et al., 2018), symbolism (Ingeborgrud and Ryghaug, 2019), and the potential for increased energy awareness through changing practices (Ryghaug and Toftaker, 2014). These endeavours are also primarily focused on the relationships between users and technology, largely eschewing the sociotechnical assemblages pertinent to automobility. Where larger sociotechnical systems and networks are included in the analysis, it is generally through the lens of MLPs (Dijk et al., 2013; Geels, 2012; Ryghaug and Skjølsvold, 2019; Skjølsvold and Ryghaug, 2020).

## Methods

In 2017 and 2018 I conducted semi-structured, open and exploratory interviews with people who drive EVs in and around Norway’s two largest cities. Invitations to participate were first circulated by email, social media and through professional and private networks. Further interviewees were subsequently found using ‘snowballing techniques’ (Atkinson and Flint, 2004), where interviewees recruited acquaintances using BEVs and PHEVs.

**Figure 2.**
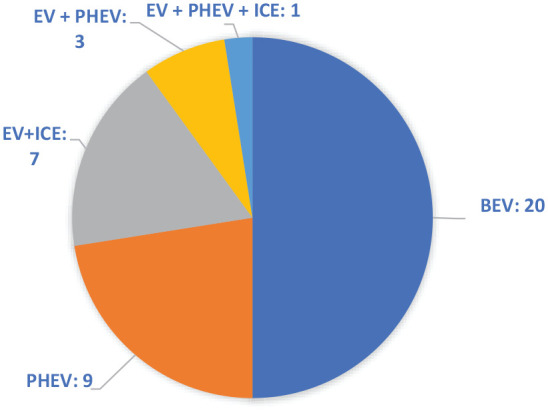
Distribution of interviewees’ vehicles.

Having been found to produce rich data on the workings of the household (Bjørnholt and Farstad, 2014), I conducted joint-couple interviews, when possible, to capture the interactions and negotiations surrounding mobility and energy use in the household. At its most productive, our joint-couple interviews resembled miniature focus groups, where partners aided recollections, created nuanced accounts and expressed diverging opinions freely. In total, 23 out of 40 interviews were couple interviews, giving a total of 63 interviewees, with ages ranging from the early 20s to late 80s. The distribution of EV, PHEV and internal combustion engine vehicles in these interviews are illustrated in Figure 2.

The interviews covered a variety of factors pertaining to EV ownership, including early experiences with EV technology, purchasing decisions, daily mobility practices, household energy use and controversies surrounding EV ownership. The interviews also broached larger topics around consumption, environmentality and lifestyle. These lasted from 45 minutes to 2 hours and were mostly conducted in the interviewees’ homes. Interviews were recorded and transcribed verbatim, and interviewees were given pseudonyms alphabetically based on the order in which they were interviewed; thus, couples interviewed together were assigned first names starting with the same letter. Inspired by grounded theory (Strauss and Corbin, 1998), the categories and codes produced in the analysis of the data were initially derived from author-generated perceptions of salient points in the discussions.

While joint-couple interviews are useful for uncovering the interactional aspects of household practice, they are not without methodological challenges. As is the case with focus group interviews, individual patterns of response may diverge from what is being presented in a collective setting (Sovacool et al., 2018). The presence of already established power dynamics in a household may also impose additional limits on what is acceptable to discuss in a joint-couple interview (Valentine, 1999). Although the topics were relatively uncontroversial, internal dynamics occasionally necessitated taking on a more active role as interviewer to ensure shared participation and facilitate open discussions, as well as applying reflexivity in the subsequent analytical process.

## Changed materiality/changing practices

While the EV is physically similar to a traditional fossil fuel vehicle and retains a multitude of the same societal functions and issues, there are considerable and marked differences in the interactions between driver and car.

Being battery powered, with a smaller driving range than a fully fuelled internal combustion engine vehicle, and with longer ‘fill’ times, matters of battery-range and charging are a critical dimension of the electric driver-car assemblage. This has led to the term ‘range anxiety’, denoting the fear of not reaching one’s destination and being stuck powerless on the road.

Participant Tormod explained the connection between changing material aspects, anxieties and concrete changes in his driving practices like this: ‘there was perhaps a small amount of range-anxiety to begin with. And then you’ll drive more softly, and not accelerate sharply to empty the battery completely every time.’ With this initial anxiety as a backdrop for Tormod’s transformed practices of driving, we can observe the co-production of technological or material change and concrete driving practice. The belief that one’s range is limited led to a more conservative driving style, limiting hard accelerations and decelerations, and discouraged extreme speeding as power consumption increases markedly in EVs when driving at higher speeds.

Other interviewees mentioned the EVs visual feedback of energy. Tree-shaped icons, developed by the Nissan Motor Co. Ltd. and built into the Nissan LEAF dashboard system, animate complex calculations of energy consumption into an easily readable and quantifiable measure. Interviewees often referred this display, or equivalent solutions from other manufacturers, as a persistent encouragement to drive economically. While visual feedback of energy use is also common in fossil fuel vehicles, my interviews established that this was a more salient concern in EVs, given the already limited range. For participant Gunnhild, this aspect of the human/non-human network represented a rather exciting way to reduce consumption: ‘It’s partly because I drive the same stretch every day, and then I’m competing with myself to conserve battery. I’m monitoring that in a way.’ As a result of this sociotechnical modification of their driver-car assemblage, both Gunnhild and Tormod reported that they were driving ‘softer’, calmer and more evenly.

Range management, visual feedback and power consumption were not the only material aspects reported as instrumental changes in the electric driver-car assemblage. Interviewees also commented on how the enhanced acceleration of electric engines affected their driving. Participant Jennifer explains:

I’m actually driving more calmly with the Tesla, because I know that I have that much power. I know that if I need to drive by someone it’s no problem. I don’t think that much about saving energy, other than perhaps on longer drives.

Jennifer considered the range available on her Tesla to be more than enough for her daily commute and thus did not feel that she had have to ‘save’ energy by adjusting her driving. Rather, she drew the line between her calmer driving style and her knowledge of the vehicle’s increased potential for acceleration.

Participants Cornelia and Christoffer also provided interesting perspectives on the physical and auditory feedback, morals and practices associated with driving their PHEV:

Christoffer: Sometimes we drive ‘til the battery runs empty, and the car switches over to gas. That’s a somewhat icky feeling.

Cornelia: It’s a very icky feeling, to be frank. And it is located within the body ….

Christoffer: I don’t know how to explain it really. It’s the sense of shame perhaps.

Cornelia: It’s a moral thing. If you are used to driving this kind of nice car, and it’s quiet, and then the fossil fuel engine turns on with all that sound and everything.

Here, we see how sound can be seen as a form of communication between the nodes in the assemblage (from the empty battery, via the engine, and to the driver’s ears and perhaps nose), and a translation made by the human actor. When Cornelia and Christoffer mismanaged range, engine noise conjured images of environmental harm, which translated into a bad conscience, feelings of environmental negligence and shame. This can be seen as an embodied manifestation of the human/hybrid-assemblage, encouraging better network management such as optimized driving practices, and a closer monitoring of remaining battery charge and more rigorous charging habits.

For participant Gunnar, it was the specificity and *otherness* of EV technology that made him reflect on his changing practices:

If someone drives past me, and accelerates faster than me, I’m just considering that I’m driving an EV. I’m no part of that at all, because I’m driving an EV. I feel as a different class of driver. Not a motorist, but an electric motorist.

In addition to noting that the technological specificity of the EV had influenced his driving, Gunnar was here also commenting on identity. After the EV entered his life, Gunnar saw himself as a different kind of driver with differing driving practices – a result of the interaction and relationship between him and his electric vehicle.

In general, calmer driving was one outcome of the electric driver-car assemblage, signifying that EV owners also may appear as safer drivers. Calmer driving practices were generally made explicit by interviewees, but these changes were characterized as embodied practices when they reflected upon their daily drives, not necessitating specific and continual consideration when the driver-car is moving. As Tormod reflected:

My driving pattern has gradually gotten more relaxed, and that is more embodied now … I’m driving more calmly and energy-saving. With my fossil fuel car, it was all about being the first one out of the traffic light.

Many of my interviewees noted the impacts on driving practices through technological changes in the driver-car assemblage − decreased range and increased fill time, increased visual feedback of energy usage and increased acceleration. This suggests that the interconnectedness of materiality and practice in the electric driver-car assemblage is also highly relevant. If anything, the connection between materiality and changing practices might even be more prevalent when looking at EV-specific practices, or as Gunnar noted: ‘It’s a closer connection between me and the engine … a shorter distance between me as human, and the engine that powers the car.’

## The electric driver-car and the world outside the windshield

While the networked interplay between battery range, enhanced acceleration and driver are crucial factors in the electric driver-car assemblage, so are factors *external* to the driver-car itself, such as topography, traffic and weather. These are also all factors pertaining to range, but physically placed outside the car itself. Being stuck in traffic eats up battery power, as does the reduced traction of rain and sleet, steep climbs and the energy necessary to keep a car’s interior comfortable during winter. The latter feature is something participant Eva, for instance, gladly sacrificed for more range. For Eva, and other interviewees, range was sometimes prioritized over comfort on cold days, while winter coats, thermoses and blankets were employed as strategies to keep bodies warm and batteries long-lasting: ‘the weather forecast says 20 below zero tomorrow. That amounts to a cold ride. … I just turn off the heat.’

From an ANT perspective, this can be seen as a material reorganizing of the network: a way for the human actor to configure different non-human actors (heater, jackets, blankets, thermoses, temperature and battery range) in relation to outside surroundings. Again, range limitations and the time required to recharge an EV makes this form of network management also appear as an EV-specific practice. Heat and range are resources with the potential to be prioritized and valued against each other.

As a consequence of this material re-configuration, Eva described driving her EV practice as ‘a completely different way of thinking’. She also added that time of day and general traffic entered into her calculations when thinking about running errands or driving to work with her EV. These changes necessitated learning, in addition to adjusting driving practice. Eva learned ‘what the car can handle’. However, as we saw with Tormod, for Eva these cognitive elements of EV operation were gradually embodied:

I think it becomes second nature. It’s not something I think about daily. I know that these are the things that’s happening today, know what the weather is, know what time it is – then I know what my day with the car will be like. It’s more automated after these years.

In addition to temperature, the more limited range and lengthier fill-times of an EV rendered geography and topography as central concerns of the electric driver-car assemblage. The capacity for longer drives, and the power used on steep inclines are clear material aspects to consider. For participant Patrik, this latter aspect influenced his driving practice, illustrating the topological variance of his daily commute:

When you’re driving down the city hillside, it’s all about generating as much power as possible …. You’re driving completely differently than you did with a gasoline or diesel-powered car. You’re looking at the regeneration constantly, ensuring it is at max.

A prominent feature of EV car technology is the potential of generating electricity through braking and managing the car’s differing eco-modes. This is especially efficient when driving downhill, where generating speed is assisted by gravity. Therefore, a new type of dance appears: The driver, brake pedal, electric engine, battery, topographical surroundings, environmental or economical motivations and the kinetic energy produced, are all moving towards the same outcome – energy regained and saved.

These assemblages, involving the drivers, cars and the driver-car’s more immediate surroundings are clearly associated with range management. There are also overarching technological overlaps informing negotiations between driver and vehicle, and between the driver-car and the outside world, like the features of battery powered propulsion, and the experienced need to conserve energy. However, it makes sense to consider the factors that are often viewed as external to the car itself as done in this section, as it captures the necessary interplay of driver-car and the world outside the vehicle. Where the driver-car historically has operated geographically in a stabilized network of gas stations and corresponding routines (Dant, 2004), these aspects of the electric driver-car assemblage are more unstable, contested and wrapped up in technological change. Range management, for instance, was often seen as something to be ‘managed’ by interviewees driving EVs with smaller batteries, but was less of a concern for interviewees with longer range EVs. Battery capacity and charging stations are essential factors to understand electric mobility and its associated practices, but these are also features in flux, as EV technology and charging infrastructure is changing, while corresponding social habits are reoriented. The proliferation of EV charging stations in Norway was sometimes reflected upon as insufficient, based on interviewees’ needs and geographic locations. Of course, other drivers, hills, valleys and varying weather have comparable impacts on the energy efficiency of fossil fuel vehicles, but currently are far more salient considerations for EV driving.

Range management was not the only significant change of the driver-car assemblage. Participant Kristina also drew upon what she perceived as the moral judgement of non-electric drivers when discussing her ambivalences as an EV driver:

I’m always conscious to let busses into my lane … signal in due time. … I feel its flat out uncomfortable to ride in the fast lane, and I must convince myself that it’s silly to contribute to the queue in the left lane.

Here, Kristina invoked a host of concerns, ambivalence and moral evaluations when explaining her discomfort when driving in the fast lane. She primarily attributed this to controversies concerning electric mobility in Norway, sometimes around conspicuous consumption, but more often around the question of EV related incentives, deemed by some non-EV drivers as unfair – for example, EV access to car lanes ordinarily set aside for busses and taxis is one of the incentives currently granted to EV owners in Norwegian cities. When reflecting upon how she had changed her driving practice, this concern was highlighted by Kristina as the most relevant change, and it seemed that the sentiments of the Norwegian anti-EV crowd had been internalized. Additionally, Kristina seemed to turn this ambivalence outward when she observed other EVs in traffic, hoping that they were behaving properly and thereby reflecting positively on the group as a whole: ‘I hope they are courteous, and let people in when they’re driving in the bus lane. That they let people through. Things like that.’ Kristina clearly identified with other EV drivers as a group – and as a part of the network – and felt both an empathic connection to, and the potential for further stigmatisation by, others in the group. Kristina’s moral calculations, and the perceived judgement of others, was an equally central feature of the electric driver-car assemblage as material and geographical facets.

Taken together, we can see changes in the relationship between human and non-human actors where fossil fuel vehicles are replaced by EVs. This includes testimonies of material changes in interplay with changing driving practice, often emphasizing calmer, energy conscious and, by extension, safer driving. Extending this perspective to how the driver-car interacts with the outside world, as done in this section, we can also see changes in how the EV is enmeshed into new assemblages of drivers, traffic, weather and topography, while practices are adjusted. As we saw with Kristina, governmental EV incentives, the reactions to these and both other EV-and non-EV drivers can also be included in these new assemblages.

In this regard, the *electric* driver-car is a new assemblage, based on a co-production of new practices brought about through the interplay between driver, technology and the outside world and represents a marked shift from the previous system of automobility.

## Networked stability and unchanging automobility

When contrasting EV specific driving practice with fossil fuel driving practice, as we did in the previous sections, some interviewees did *not* regard this to be a meaningful dichotomy. Rather, they considered the features of their EV to be a function of the newness of the car in general. When discussing driver assistance systems, enhanced energy management and other technological changes, these were not understood as features specific to EVs. Participant Fredrik, for instance, felt that there were no marked changes in his driving practices after acquiring an EV, and questioned whether the newer technological features of his vehicle were indeed specific to EVs: ‘Driving does not feel any different, but just being in an EV feels different. You press a button rather than gearing, and stuff like that. Or, it’s starting to be like that in other cars as well, I think.’ Several of the interviewees reflected positively on the development of pre-heating cars in the morning, having app control, Bluetooth and other technological conveniences, these appeared more to be consequences of their car being new, rather than novel factors specific to EVs. Further, many noted that while these features were initially appealing, they quickly lost relevance. For participant Roger, for instance, his new Tesla soon turned into just an ordinary car: ‘In the beginning it was a bit extraordinary to have an EV, or a Tesla to be more specific, but it only took 1 year for it to not feel special any more. It’s just a car, nothing more.’ Taken together with the previous paragraph, these accounts raise the question of how novel the electric driver-car assemblage actually is, and how much credence should be given to the accounts of EVs radically upending the established system of automobility.

Central to Urry’s (2004) characterization of the automobility system is its autopoietic qualities, that is, how the system is continuously reproducing itself. The broad societal changes of automobility are widely acknowledged, and Urry’s point is that these changes have made us even more dependent on the car. Interviewing EV users, it is clear that the proliferation of EVs and EV specific practice does not necessarily challenge this dependency.

If anything, some interviewees reported using their EVs *more* than they used to drive their fossil fuel vehicles. As participant Mathilde explained:

It feels bad to admit that I might drive more now than what I previously did. It’s like my conscience is better when driving now, so I use the car more. I’ve become lazier, because it doesn’t harm, I don’t fill gas and it’s almost free. So that’s good economic and environmental conscience, and it’s also cheaper than taking the bus. I think I’m driving more when it’s not necessary. If I were to drive a diesel, I would probably consider it more. I would rather consider walking, taking the bus or something like that.

Again, the matter of moral evaluation and feelings related to the act of driving is raised. More environmentally focused interviewees reflected often on how they were now driving with a better conscience in an EV and for some this resulted in using the car more. Where environmental concerns were no longer directly experienced, this sometimes resulted in more frequent car trips. We can also see the economic aspects of EV driving factoring into this proclivity to drive more. In Norway, electricity is far cheaper than gasoline or diesel, and economic incentives for EV drivers have been a significant driver of EV proliferation in the country (Bjerkan et al., 2016; Figenbaum, 2017; Fridstrøm and Østli, 2017; Ryghaug and Toftaker, 2014). Jennifer, for instance, felt that there was a direct link between the elimination of toll road payments for EV drivers and her driving more:

I’m driving through the city toll when I go to work out, and that was 30 NOK extra each time. And now that does not exist for me so it’s easier to take an extra trip. Also, you don’t need to plan your trips to do as much as possible on every run, it’s easier to just pop out.

In addition to increasing trip frequency, some interviewees reported an increased preference to use the EV over other modes of transportation, like walking, cycling and public transportation. Participant Olivia noted:

Too be sure, I’ve become lazier after we got the EV. Just because I feel like I can drive with a clear conscience, I’m driving everywhere. Even if we’re going to the local store that is only a couple of hundred meters away. I’m also using the bus less than what I did before, or what I would do if we still had the large diesel car. … We were better at taking the bus, and better at walking.

As these accounts show, matters of economy, conscience and convenience are concerns in the electric driver-car assemblage, which in some cases may lead to less desirable outcomes such as increased car use. Thus, rather than representing a transition from the entrenched system of automobility, the introduction of EVs may, at best, reinforce, and, at worst, strengthen the system. Here, the relationship between driver and technology are stable or strengthened, the cars are still understood as ‘just a car’ and the continued reliance on individual automobility becomes practical, environmentally palatable and cheaper with an EV.

## The rural network nodes, and the car(s) as work horse

This recounting of the stabilization of the automobility system would, however, be incomplete without accounting for the perceived need for cars, and how integrated cars are in the interviewee’s lives.

Many interviewees owned a cabin and the specific range required to reach these cabins was a significant factor when purchasing a car. The Norwegian cabin tends to be located in rural areas, not always with readily available charging options on-site or along the way. Distances to cabins were often identified as the primary reason why interviewees acquired long-range electric vehicles, PHEVs or kept a fossil fuel vehicle as ‘backup’. For participant Ada, for instance, this was a salient issue:

We travel to the south of Norway a couple of times a year, so we need a car with long range, even if we charge a couple of times along the way. And we have always had one car, and we will not have two cars. That is my reasoning for acquiring a Tesla, on account of it being the only car with long range.

Again, moral calculations regarding consumption and environmentality appeared relevant, as it was important for Ada and her partner to maintain a one-car household. While this had invited accusations of conspicuous consumption from friends and co-workers, as their Tesla Model S was considered both flashy and expensive, they felt that this car was the most environmentally sound way to enable their vacation trips. The matter of maintaining a one-car household while still reaching their cabin was also important for Christoffer and his partner. Their choice was, however, a PHEV:

We started testing out EVs. Started with a Nissan LEAF …. But then we felt that we had a range issue, perhaps. We have a cabin that’s 320 km away, with scant charging options along the way. So that amounted to a range issue, in addition to wanting a tow bar, roof luggage box, and stuff like that. So, we found that the second-best thing, when we could not afford a Tesla, was a chargeable hybrid.

As we see in both Ada and Christoffer’s account, the cabin, and the Norwegian cabin culture, is a cogent factor in many electric driver-car assemblages, either on account of the specific EV chosen, or the choice to keep a backup fossil fuel vehicle for longer drives.

Cabins were not the only thing motivating these backups, as Christoffer touched upon. Participants Frida and Fredrik, for example, chose to ‘outsource’ the studded tires to their old internal combustion engine Mercedes, maintaining a somewhat environmentally clear conscience until the roads freeze over:

Frida: We choose to not have studded tires on the EV.

Fredrik: But then again, we have that on the Mercedes.

Frida: Then we have an option on the days we actually need studded tires to move. There’s no discussion of environmental harm on those days, because then it’s just ice.

Fredrik: And there’s also ice [on the road when] driving to the cabin during winter. Then I feel safer with the claws out.

Here, we see an interaction between road conditions, route, choice of car and choice of tires, and how safety and environmentality can be valued. This ‘outsourcing’ was also common when retaining a tow-bar, a feature currently rare in EVs, but sought-after by Norwegian car owners. When asked why they kept their old and rarely used diesel, Gunnhild and Gunnar quickly responded:

Gunnar: Trailer! We have a boat that often needs to go on a trailer

Gunnhild: And we have a house with a garden, and things that need to be moved away, so we have the sporadic need of that.

Many interviewees valued the capacity to pull a trailer as an integral part of their mobility network. We can also see the wish for features more commonly associated with fossil fuel vehicles in Kristina’s lamenting the lack of four-wheel drive on her Tesla for navigating her daily commute, and the drive to the family cabin.^1^ ‘It bothers me that it doesn’t have four-wheel drive. Living in the city hillside, and travelling to my parents’ cabin in the mountains, we need a four-wheel.’ For many, the solution was to retain their old car as a backup, which enabled the network to function as it previously did.

This negotiated entourage of primary vehicles, backups, trailers, boats and cabins are not necessarily stable in-and-of-themselves, but rather shift as the human actors gain experience with the technology. What was initially intended as a cheap, secondary city-car can suddenly become the primary vehicle of the household. As participant Lars explained: ‘We planned for the EV to be the secondary car. But then it became apparent … it’s car number one, right? And the other car is just in use when we’re travelling to the cabin and longer.’ Tales of oft-neglected ‘daily drivers’ were not uncommon, and Fredrik noted that he now used his previously beloved internal combustion engine Mercedes so rarely that *its* battery often ran out of juice between drives. The main takeaway, however, is that the EVs are, in these cases, not replacing aspects of the existing mobility network, but rather adding to them, with more vehicles of varying practical functions.

Specific practices such as travelling to rural cabins or hauling trailers appear as stable nodes in the Norwegian mobility system. If the EV is unable reach the cabin, drivers opt for one that will, or choose to keep the old fossil fuel vehicle. As EV technology continues to change, while battery capacity and practical and technical features are added, there will be more EVs fitting these criteria. From this perspective, EVs are, or will be, compatible with the cultural, material and geographical prerequisites of the established networks, rather than engendering systemic change.

Again, we can draw upon Urry’s observation on the autopoietic qualities of the automobile system, and the observation that automobility has already created a framework for the continued proliferation of the system (Urry, 2004). Analysing changes in the Norwegian mobility system, it seems clear that the proliferation of EVs does little to upend the established system. Rather than changing the patterns of mobility enabled by automobility, the EV is in this perspective enabling established movement patterns and corresponding practices and desires. Further, the EV is tailor-made for the established transportation system and benefits from its prevalence and obduracy. EVs provide many of the same conveniences as fossil fuel vehicles and as EVs gain capacity and new features, they synchronize with established user expectations of what a car can and should do. The more that EVs are ‘normal cars’ in capacity and aesthetics, and the better they serve already established patterns of mobility, the more appealing they appear. The automobility regime, following MLP parlance, is in this perspective not a barrier to a beneficial and disruptive EV niche, but rather constitute the prerequisite condition that EV technology seeks to serve.

## Between stability and change

MLP has proved particularly productive when providing a sociotechnical mapping of the transition towards electromobility. However, an overemphasis on the transformative potential of the niche (Geels, 2018) and a proclivity for bottom-up change models (Sovacool and Hess, 2017), makes the theory also appear less equipped for studying the relational aspects of the automobility system.

It is here that a re-orientation towards the interplay of human and non-human actors in the electromobility network proves useful. This article shows how analysis of the networks formed around the electric vehicle, and their related practices, reveals new developments while at the same time uncovering reasons for pause. On one hand, I observed exciting interactions when novel technology enters new networks and generates entanglement with the sociotechnical and natural world. Also, I analysed the negotiations between artefact and owner, where new practices are formed and new meanings are constructed. These are stories of how the engine noise of a poorly charged, or inefficiently operated, plug-in hybrid can translate into feelings of shame, how smaller batteries or increased acceleration influence driving practices and how a heated interior is traded for blankets and jackets for optimum driving range during winter. We see brake pedals, generators, hillsides and drivers pulling together to regain energy, and the new narratives of drivers feeling a closer connection to their vehicles. Rather than associating change with the specific niche being analysed, and the venues of politics, markets and technological development enabling the niche and opening windows of opportunity, ANT serves to highlight how change is produced in the interplay between users, technology and the surrounding natural and man-made landscapes. By investigating how EVs become an element in human/non-human entanglements, we can analyse the new associations formed, and their potential for change.

However, and more crucially, an ANT-inspired approach to mobility transitions also provides the framework to understand aspects of the automobility system that do not change as EVs gets more prevalent. These are narratives of previously established and unchanged transportation needs, of cabins, boats and trailers putting specific and non-negotiable requirements on EVs, and the old fossil fuel vehicle that keeps its place in the garage. This includes accounts of the EV being ‘just a car’, still taking us back and forth between home and work and other mundane elements of our everyday lives, and the pervasive negative consequences of automobility that remain while the technology changes. Here, against prevalent niche optimism, ANT appears as a particularly fruitful approach to study the stability of these obdurate networks.

While some specific aspects of the sociotechnical network analysed in this article may be particular to the Norwegian context – such as Norwegian cabin culture and specific economic incentives – aspects of this case can be generalized. Range, for instance, regains relevance and complexity in the interviews and the subsequent analysis. Where an MLP-influenced understanding might argue that technological niche developments reduce range-anxiety as EVs gain more range potential, ANT-oriented inquiries supplement this perspective by analysing relationships between drivers, vehicles and the world outside the windshield. Range is not solely determined by the capacity of the battery itself, but also as the outcome of networked processes between users, technologies, driving practices, cultures, weather and topography – a shift in perspective indeed transferable to other contexts.

The challenges and potential pitfalls of mapping conceptual levels (landscapes, regimes and niches) onto empirical findings was an early critique of MLP-approaches (Berkhout et al., 2004). While this is a matter of analytical levels, findings in this article cast doubt on whether conceptualizing EVs as disruptive technology is the best point of departure. Rather, it may be more constructive to also consider the conserving potential of the technology, and how it facilitates ‘the preconditions for its own self-expansion’ (Urry, 2004: 27).

However, viewing the material and practical changes of EV technology solely through the lens of the automobility system may make us less sensitive to changes occurring between driver, technology and the larger sociotechnical systems. Here, the concept of energy citizenship, and how the adoption of energy technology may engender material participation (Ryghaug et al., 2018), appears particularly useful. EVs may not bring about disruptive and systemic change to the obdurate automobility regime, but research has already explored the relationship between the technology and pro-environmental attitudes, perspectives on identity and gender, in addition to changing practices of driving (Anfinsen et al., 2018; Ingeborgrud and Ryghaug, 2017; Ryghaug and Toftaker, 2014).

In summary, EV technology, and the electric mobility transition, is fraught with tensions and somewhat still in flux. Somewhere between overzealous claims of revolutionary advances and business as usual, between new practices and previously established patterns of mobility, between ‘iron cages’ and possibility spaces – and somewhere between stability and change.

## Notes

1. Since this interview, Tesla has implemented four-wheel drive in all their vehicles, further illustrating how EVs are gradually gaining features and becoming more similar to fossil fuel vehicles. Additionally, the quick development of technological changes clearly illustrates how EV technology is still in flux.
